# Martha Wheelock

**Published:** 1899-12

**Authors:** 


MARTHA WHEELOCK, wife of Dr. C. W. Bourne, of Hamburg, died November 14, 1899, of typhoid pneumonia, after a very short illness. Mrs. Bourne was a daughter of the late Silas B. Wheelock, one of the pioneers of this region, and was married to Dr. Bourne soon after the close of the Civil War. She leaves one daughter, Mrs. George Potter, and two sons, Dr. B. S. Bourne and Charles Bourne, of Hamburg. Mrs. Bourne was a member of the Hamburg M. E. Church, the Woman's Christian Temperance Union and several other organisations. Dr. Bourne is one of the most prominent physicians in Erie County and will receive the sympathy of a great community.

